# Active Chronic Hepatitis B increases the risk of Colorectal Liver Metastasis - A retrospective cross-sectional study

**DOI:** 10.7150/jca.51233

**Published:** 2021-01-01

**Authors:** Yue Yang, Lijie Song, Jingyu Cao, Jing Liu, Dongxu Wang, Linda L. Wong, Lei Zhao

**Affiliations:** 1 Shandong First Medical University and Shandong Academy of Medical Sciences, 6699 Qingdao road, Huaiyin District, Jinan, China.; 2 Department of Hepatobiliary Surgery, Shandong Cancer Hospital and Institute, Shandong First Medical University and Shandong Academy of Medical Sciences, 440 Jiyan road, Huaiyin District, Jinan, China.; 3 Department of Oncology, The First Affiliated Hospital of Zhengzhou University, 2 Jianshe East Road, Erqi District, Zhengzhou, China.; 4 Department of Hepatobiliary Surgery, The Affiliated Hospital of Qingdao University, 1677 Wutaishan Road, Huangdao District, Qingdao, China.; 5 Department of Biostatistics, School of Public Health, Shandong University, 44 Wenhua West Road, Lixia District, Jinan, China.; 6 Department of Liver Surgery, Peking Union Medical College Hospital, Chinese Academy of Medical Sciences and Peking Union Medical College, No.1 Shuaifuyuan Wangfujing Dongcheng District, Beijing, China.; 7 Department of Surgery, University of Hawaii School of Medicine, 3430 Keahi Place, Honolulu, HI96822, USA.

**Keywords:** chronic hepatitis B (CHB), colorectal liver metastasis (CRLM), hepatitis B e antigen (HBeAg), risk

## Abstract

**Background:** A considerable part of colorectal cancer (CRC) patients also have chronic hepatitis B (CHB), esp. in Asia. The effect of concomitant active CHB on the hazard of colorectal liver metastasis (CRLM) remains unclear. To evaluate the effect of concomitant active CHB on the risk of CRLM.

**Methods:** The medical record of all newly diagnosed CRC patients who were hospitalized to the three hospitals between January 2010 to January 2016 were reviewed, the prevalence of synchronous CRLM (synCRLM) were retrospectively studied. Totally 7187 cases of newly diagnosed CRC, including 368 cases with concomitant CHB were recruited. The prevalence of synCRLM in HBsAg^+^/HBeAg^+^ patients was compared to that in HBsAg^+^/HBeAg^-^ patients. Significant risk factors for synCRLM were analyzed by logistic regression analysis.

**Results:** The overall prevalence of synCRLM was 8.72% (627/7187) and was significantly higher in HBsAg+ patients (43/368) than HBsAg- patients (576/6742) (11.68% vs. 8.54%, P=0.037; χ^2^ test).In 368 HBsAg^+^ patients, 365 patients also had HBeAg information. synCRLM was also more prevalent inHBsAg^+^/HBeAg^+^ patients (13/69) compared to HBsAg^+^/HBeAg^-^ patients (30/296) (18.84% *vs.* 10.14%, *P*=0.043; *χ*^2^ test). In univariate and multivariate logistic regression analysis, HBeAg positivity was the second strongest predictor of synCRLM (multivariate: OR, 2.622, *P*=0.020) after CEA. (univariate: OR, 2.920, *P*=0.001).

**Conclusions:** HBeAg positivity is a clinical risk factor for CRLM that can be readily identified and addressed. Whether anti-CHB treatment can decrease the risk of CRLM worth carefully-designed prospective trials to define.

## Introduction

Worldwide, colorectal cancer (CRC) is the fourth primary reason of cancer mortality and is increasing in medium to highly-developed countries especially in Asia and Europe. China has the highest number of new cases, accounting for about 28% of global CRC cases 1, 2.

Asia also has a heavy burden of viral hepatitis B. It is estimated that the global prevalence of hepatitis B surface antigen (HBsAg) was 3.9% in 2016 3. Among 9 Asian countries which each had more than 10,000 new CRC cases in 2018, the prevalence of HBsAg in China, Indonesia, Philippines and Vietnam were much higher than the average global level, while the HBsAg prevalence in India, Republic of Korea, Turkey, Thailand varied between 2.4 and 3.5% 4. Although hepatitis B virus (HBV) prevention policy in China has significantly decreased the prevalence of HBsAg in the younger population, HBV infection remains endemic in those over 40 years of age 5.

As a consequence, many CRC patients in Asia also have chronic hepatitis B (CHB) infection. It has not been clearly expounded that the effect of concomitant CHB infection on the risk of colorectal liver metastasis (CRLM). Quite a number of clinicians believe that CHB has a “protective effect”. Although previous reports support their hypothesis, these studies were limited by small sample size, mixed types of chronic hepatitis and xenotherapy 6. Our previous study showed that CHB-induced liver cirrhosis is anti-metastatic, but failed to prove that active CHB, represented by positive Hepatitis B e antigen (HBeAg) is pro-metastatic 7. This current study expands upon our experience with a larger sample size and hopes to further address this issue.

## Materials and Methods

### Data Source

We performed this retrospective, cross-sectional study of 7187 consecutive newly diagnosed CRC cases in three hospitals. The Ethics Committee of whole three hospitals permitted this study. All patients were hospitalized during the period between January 2010 and January 2016 and had pathological confirmation of their CRC.

Retrospective data collection involved demographic information such as age, gender and laboratory studies, containing the complete blood counts, liver function tests and HBV specific tests. Liver fibrosis/cirrhosis was measured and calculated by the indicator that aspartate aminotransferase (AST) to platelet ratio index (APRI).According to the American Joint Committee on Cancer (AJCC) staging, tumor characteristics, involving tumor size, differentiation (well, moderate or poor) and tumor (T) and nodal stage (N), were also noted based on pathology reports when available, or otherwise on imaging reports.

The definition and diagnosis of synchronous colorectal liver metastasis (synCRLM) was same as our preceding report 7. SynCRLM was defined as the simultaneous diagnosis of liver metastasis together with primary CRC; in brief, all patients with CRC underwent chest radiography and abdominal and pelvic ultrasonography (US). If these studies suggested possible pulmonary and/or hepatic lesion(s), computed tomography (CT) scan was performed to confirm the diagnosis. All liver metastases were verified with CT scan that was reviewed independently by two senior radiotherapists. SynCRLM was defined as the simultaneous diagnosis of liver metastasis together with primary CRC.

This study was divided all patients into different groups depending upon the status of HBsAg (HBsAg^+^ *vs.* HBsAg^-^) or HBeAg (HBeAg^+^ *vs.* HBeAg^-^). Baseline clinicopathological parameters and the prevalence of synCRLM were compared between these groups.

### Inclusion and exclusion criteria

The inclusion criteria are as followings: (1) all pathological sections that were primary adenocarcinomas in the colon and rectum; (2) no treatment before admission; (3) diagnosed of CRC based on both the postoperative pathology and the electronic colonoscopy biopsy; (4) had relatively complete medical data records.

The exclusion criteria are as followings: (1) patients who have had other treatments, not newly diagnosed or treated; (2) without pathological diagnosis; (3) with other concomitant malignancies.

### Statistical analysis

The prevalence of synCRLM in different groups was compared by chi-square (*χ*^2^) test. Baseline categorical clinical parameters were compared by *χ*^2^ test, and numerical values were compared using Student's *t* test. APRI were compared by Wilcoxon rank sum test. Significant risk factors for synCRLM were analyzed first by univariate logistic regression analysis and then by multivariate logistic regression analysis. Statistical analysis was performed by SPSS17.0.

## Results

**Figure 1:**
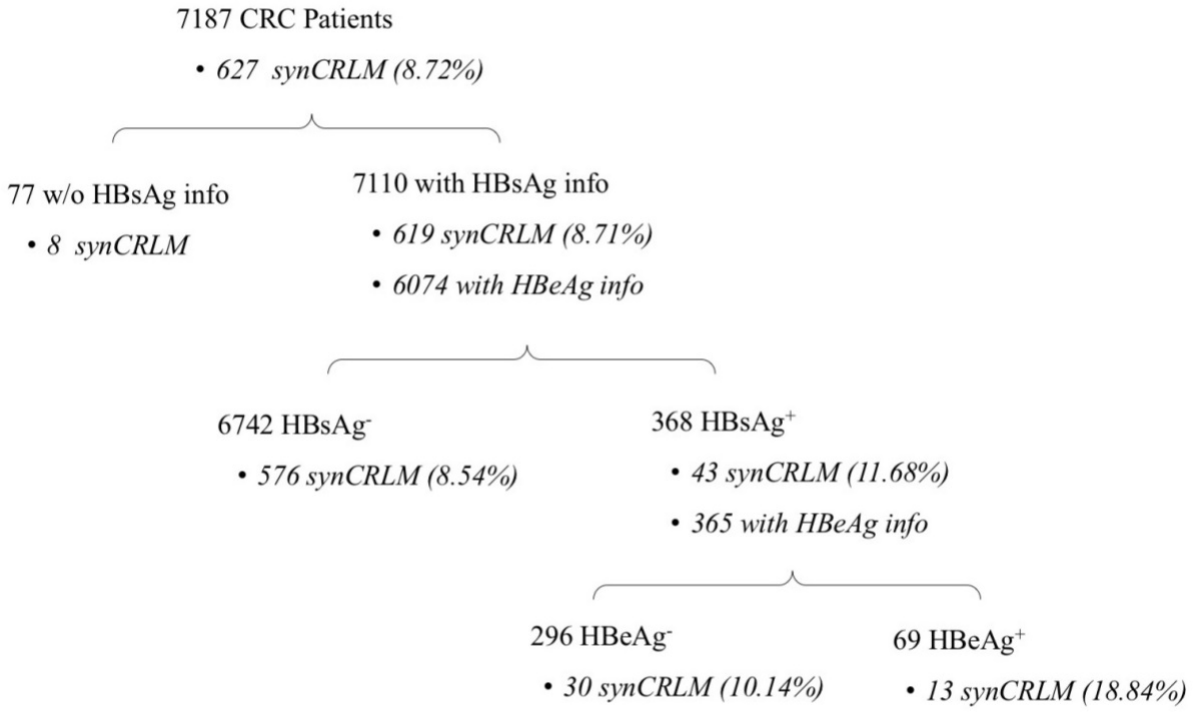
Number of patients with HBsAg/HBeAg positivity and synCRLM prevalence.

Patients' information about HBV infection and synCRLM prevalence are presented in **Figure 1.** In the entire cohort of 7187 CRC patients, 7110 patients had the information of HBsAg test, the prevalence of HBsAg positivity was 5.18% (368/7110); Baseline clinicopathological parameters of the HBsAg^+^ and HBsAg^-^ cohorts are presented in **Table 1.** The mean age of the HBsAg^-^ patients was significantly higher than that of the HBsAg^+^ patients (mean±SD, 60.69±12.81 *vs.* 56.66±11.61 years, *P*=0.001).

The mean carbohydrate antigen 19-9 (CA19-9) value in HBsAg^+^ patients was also slightly higher than that in HBsAg^-^ patients but achieved statistical significance (55.81±144.66 *vs.* 55.64±235.67, *P*=0.001). No statistical differences were found among tumor size or stage, gender distribution and carcinoembryonic antigen (CEA).

Complete blood counts and liver function parameters of the HBsAg^+^ and HBsAg^-^ cohorts are also presented in **Table 1.** White blood cell (WBC), neutrophil, eosinophils and platelet counts in HBsAg^+^ patients were significantly lower than those of the HBsAg^-^ patients, these can be explained by the CHB-related liver fibrosis/cirrhosis and hypersplenium, as the APRI value in the HBsAg^+^ patients was significantly higher than in the HBsAg^-^ patients (0.370±0.635 *vs.* 0.225±0.477; *P*=0.001; **Table 1**). Also, in the HBsAg^+^ patients, their liver tests were significantly worse compared to the HBsAg^-^ patients, indicated by the elevated aminotransferase, bilirubin and decreased globulin (GLB), prealbumin, triglyceride (TG), cholesterol (CHOL) and Low Density Lipoprotein (LDL).

The overall prevalence of synCRLM was 8.72% (627/7187). Moreover, the cohort of HBsAg^+^ patients (43/368) was significantly higher than that of HBsAg^-^ patients (576/6742) (11.68% *vs.* 8.54%, *P*=0.037; *χ*^2^test; **Table 1**).

Based on the chief complains and the history of present illness in the medical record, the initial manifestations included bleeding or blood in stool; change in the consistency, size or shape of stools; change in frequency of bowel movements; abdominal pain or discomfort; abdominal mass. The most common initial manifestation was bleeding or blood in stool. Both HBsAg^+^ and HBsAg^-^ patients showed that no significant difference in the distribution of initial manifestation were observed (*P*=0.274; *χ*^2^ test, **Table 1**). Only 6 out of 7187 patients were diagnosed by a routine asymptomatic colonoscopy examination, they were all HBsAg^-^ and had no synCRLM; If these 6 patients were excluded, synCRLM remained more prevalent in HBsAg^+^ patients (43/368) compared to HBsAg^-^ patients (576/6736) (11.68% *vs.* 8.55%, *P*=0.038; *χ*^2^ test, **Table 1**).

In 7110 patients with HBsAg information, 6074 patients had HBeAg test performed, and HBeAg positivity was noted in 1.14% patients (69/6074). In 368 HBsAg^+^ patients, 365 patients also had HBeAg information. Baseline clinicopathological parameters of the HBsAg^+^/HBeAg^+^ and HBsAg^+^/HBeAg^-^ cohorts are presented in **Table 2.** Male domination was significant in HBeAg^+^ cohort (69.57% *vs.* 55.74%, *P*=0.036). The CA19-9 value in HBsAg^+^/HBeAg^+^ patients was significantly higher than that in HBsAg^+^/HBeAg^-^ patients (100.22±221.79 *vs.* 45.17±117.24, *P*=0.001). There were no significant differences in age, tumor size or stage and CEA.

Complete blood counts and liver function parameters of the HBsAg^+^/HBeAg^+^ and HBsAg^+^/HBeAg^-^ cohorts are also presented in **Table 2.** Here only neutrophil and platelet counts were significantly lower in the HBsAg^+^/HBeAg^+^ patients compared with HBsAg^+^/HBeAg^-^ patients, and double positive patients did have significantly higher APRI value as well as aminotransferase, GGT and ALP, but not bilirubin. These results indicated that the HBsAg^+^/HBeAg^+^ cohort is in general more fibrotic or cirrhotic.

In 365 HBsAg^+^ patients with HBeAg information, 19 HBsAg^+^/HBeAg^+^ patients and 40 HBsAg^+^/HBeAg^-^ patients showed elevated ALT, while only 2 of them were under the anti-HBV treatment. There were other 7 patients with normal ALT are under or had a history of anti-HBV treatment. The anti-HBV treatments includes: 1 with interferon-α, 1 with lamivudine, 2 with adefovir, 2 with lamivudine plus adefovir and 3 with entecavir.

In further sub-group analysis, synCRLM was also more prevalent in HBsAg^+^/HBeAg^+^ patients (13/69) compared with HBsAg^+^/HBeAg^-^ patients (30/296) (18.84% *vs.* 10.14%, *P*=0.043; *χ*^2^ test, **Table 2**). If 9 patients with anti-HBV treatment were excluded, synCRLM remained more prevalent in HBsAg^+^/HBeAg^+^ patients (13/67) compared to HBsAg^+^/HBeAg^-^ patients (30/289) (19.40% *vs.* 10.38%, *P*=0.032; *χ*^2^ test, **Table 2**).

In univariate logistic regression analysis, the odds ratio (OR) of HBeAg positivity was the highest [OR: 2.920, 95% confidence interval (CI): 1.588-5.371, *P*=0.001; **Table 3**], which was more than twice that of HBsAg positivity (OR: 1.417, 95% CI: 1.019-1.969, *P*=0.038; **Table 3**). In the subsequent multivariate analysis with other significant factors, HBeAg positivity remained the second strongest predictor of synCRLM only after CEA. (OR: 2.622, 95% CI: 1.164-5.903, *P*=0.020; **Table 4**), the OR of HBsAg positivity was 1.565 (95% CI: 1.009-2.427, *P*=0.046; **Table 5**).

## Discussion

In our previous study of 4033 CRC patients, contrary to much of the reported literature, positive HBsAg was associated with a significant increase in the prevalence of CRLM. Patients with active viral replication as determined by HBeAg positivity also trended toward increased CRLM prevalence but did not quite reach statistical 'significance 8. In this study, we used the same criteria to recruit 3154 patients from the First Affiliated Hospital of Zhengzhou University as a third cohort. HBsAg+/HBeAg^+^ patients showed significantly higher APRI value, AST, GGT and ALP as well as decreased neutrophil and platelet counts, suggesting that they were generally more fibrotic compared to HBsAg^+^/HBeAg^-^ patients. Our previous study demonstrated that CHB-induced liver fibrosis/cirrhosis was anti-metastatic, as an increased APRI was associated with lower risk of CRLM 9. Despite this, HBsAg^+^/HBeAg^+^ patients had significantly higher prevalence of synCRLM. By univariate and multivariate logistic regression analysis, HBeAg positivity was an independent predictor of CRLM with higher odds ratio than HBsAg positivity.

According to the AASLD (American Association for the Study of Liver Diseases) 2018 Hepatitis B Guidance, CHB patients with elevated ALT should be evaluated with other tests (HBV-DNA, HBeAg, etc.) to determine the need for treatment with antiviral agents 10. In our study, 59 HBsAg^+^ patients showed elevated ALT, but only 2 of them received anti-HBV treatment. In China, only 19% of CHB patients are diagnosed and only 10-11% of eligible CHB patients receive anti-HBV treatment 11. Only very limited number of CHB patients in our study received or are currently receiving anti-HBV treatment, excluding these patients from the analysis would not affect the results and synCRLM remained more prevalent in HBsAg^+^/HBeAg^+^ patients. Future studies with a larger proportion of treated patients separately analyzed would be needed to observe the true effect of antiviral agents on the risk of CRLM.

Although the pathogenesis of how HBV influences CRC is not completely clear, there are potential mechanisms that should be considered. Virologists have discovered that active HBV replication raised the expression of a group of chemokines, including CCL20, CXCL6 and the CXCL9/10/11 family. Notably, these chemokines showed a stepwise increase from healthy individuals to asymptomatic HBV carriers and then to patients with CHB 12. Oncologists and gastroenterologists have developed substantial evidence that these same chemokines increase the risk of CRLM 13. To our knowledge, no study has linked these concepts to propose a mechanism for CRLM pathogenesis in the face of HBV. We are currently exploring this further by performing chemokine screening on CRLM patients to better delineate the relevant chemokines and their mechanisms.

Unlike the minimal chance of becoming HBsAg negative, HBV-DNA suppression and the loss of HBeAg can be achieved in many patients with CHB using current anti-viral therapies 14. HBeAg may be a more useful marker in CRC patients. If reduction in viral replication and infectivity can be achieved with anti-viral agents in patients, this may be potentially beneficial for decreasing CRLM and prolonging life in CRC patients who also have HBV.

This study is limited in that it is a retrospective cross-sectional prevalence study; however it would be impossible to study synCRLM in a prospective manner. A prospective study of all CRC patients to observe for metachronous CRLM stratified for HBV and with measurement of various chemokines would be a theoretical way to understand this. However, such a study would be practically too complex to conduct because it would require long-term follow up and the identical parallel use of neoadjuvant and adjuvant chemotherapy, newer targeted therapy and immunotherapy plus the use of antivirals for HBV. There would be too many variables which may affect each other to understand the true effect of HBV on development of CRLM.

Screening colonoscopy can reduce the incidence of CRC by identifying and removing precancerous polyps and by prompting an increase in the surveillance frequency. Unfortunately, China does not currently have a nationwide CRC screening or surveillance programs with colonoscopy. Awareness and education on CRC in most of China is generally poor, only very limited individuals with better education and socioeconomic status privately pay for the screening colonoscopy examinations. Even among high-risk populations of CRC in urban China, where the education is considered to be better than rural areas, the compliance rate for freely-provided colonoscopy was only 15.3% 15.

China's hospitals do not have centralized electronic medical record system, instead, every hospital use its own hospital information system (HIS) and medical record system (MRS). As the consequence, we can't check whether these patients had previous colonoscopy examinations or not. Based on the chief complains and the history of present illness in the current medical record, we meticulously checked the initial manifestation of all 7187 patients, and discover that only 6 patients were diagnosed by a routine asymptomatic colonoscopy examination, exclusion of these 6 patients does not affect the statistic results of the prevalence between HBsAg^+^ and HBsAg^-^ cohorts. Therefore, it is unlikely that the use of screening colonoscopy presented a potential bias between the groups.

As the burden of CRC continues to rise in countries in which CHB is endemic, serologic screening for CHB in newly diagnosed CRC should be advised. Screening should also be considered in non-endemic areas if patients have risk factors for HBV and their viral status is not known. Antiviral agents are typically given to prevent the dangerous consequences of HBV reactivation while on chemotherapy. Patients who have relatively advanced CRC (stage IIb or higher) will likely receive neoadjuvant or adjuvant chemotherapy and those patients who also have concomitant CHB should have anti-HBV therapy initiated. However, as CRC screening programs have increased the detection of early stage CRC patients 16, it will be important to develop strategies for prevention of CRC progression. Early stage CRC patients may undergo only surgical resection but are still at risk for developing CRLM in the future. The 5-year cumulative metachronous CRLM rate was reported to reach 3.7% and 13.3% for TNM stage I and II CRC, respectively 17. If the association between active CHB and the risk of CRLM is validated with other studies, it will be essential to determine the optimal candidate for HBV therapy in terms of liver function and stage of fibrosis/cirrhosis, the timing/length of therapy and surveillance for CRLM.

This study has limitations. First, we lacked HBV information of some patients, so the missing data may cause information bias. Then, the sample size is limited, and the research method is vulnerable to the influence of unbalanced quality method. Finally, we believe that future research can continue to focus on the prevention and efficacy of CRLM after antiviral clinical treatment.

## Conclusions

In summary, HBeAg positivity is a clinical risk factor for CRLM that can be readily identified and disposed. It is yet unclear if antiviral treatment can decrease the risk of liver metastasis in CRC patients, but future studies with carefully designed prospective trials will be needed to better define this.

## Figures and Tables

**Table 1:** Baseline clinicopathological of HBsAg^+^ and HBsAg^-^ cohorts

Factors	HBsAg^+^ (N=368)	HBsAg^-^ (N=6742)	*P*
synCRLM (Yes/No)	43/325	576/6166	**0.037**
Gender (male/female)	214/154	3975/2767	0.759
Age (years, mean±SD)	56.66±11.61	60.69±12.81	**0.001**
**Initial manifestation**			
Bleeding or blood in stool	93 (25.27%)	1702 (25.24%)	
Change in the consistency, size or shape of stools	18 (4.89%)	275 (4.08%)	
Change in frequency of bowel movements	11 (2.99%)	277 (4.11%)	
Abdominal pain or discomfort	42 (11.41%)	1103 (16.36%)	
Abdominal mass	2 (0.54%)	34 (0.50%)	
N/A or other initial manifestation	202 (54.90%)	3345 (49.62%)	
Asymptomatic colonoscopy exam (ACE)	0	6 (0.09%)	**0.274**
synCRLM excluding ACE (Yes/No)	43/325	576/6160	**0.038**
**Primary CRC**			
Tumor size (cm)	4.71±2.00	4.74±2.04	0.852
**Location**			
Colon	128 (34.78%)	2357 (34.96%)	
Rectum	129 (35.06%)	2581 (38.28%)	0.516
N/A	111 (30.16%)	1804 (26.76%)	
**Grade**			
Poorly	59 (16.03%)	961 (14.25%)	
Moderately	256 (69.57%)	4638 (68.79%)	
Well	13 (3.53%)	235 (3.49%)	0.772
N/A	40 (10.87%)	908 (13.47%)	
**T stage**			
Tis-T2	55 (14.95%)	1088 (16.14%)	
T3- T4	271 (73.64%)	4634 (68.73%)	0.336
N/A	42 (11.41%)	1020 (15.13%)	
**N stage**			
N0	194 (52.72%)	3440 (51.02%)	
N1-N2	127 (34.51%)	2211 (32.80%)	0.876
N/A	47 (12.77%)	1091 (16.18%)	
CEA (ng/ml)	28.21±109.65	27.74±106.34	0.557
CA199 (U/ml)	55.81±144.66	55.64±235.67	**0.001**

HBsAg: hepatitis B surface antigen; synCRLM: synchronous colorectal liver metastasis; N/A: not available; Tis: tumor *in situ*; CEA: Carcinoembryonic Antigen; CA199: Carbohydrate Antigen 19-9.

**Table 2:** Complete blood counts of HBsAg^+^ and HBsAg^-^ cohorts

Factors	HBsAg^+^ (N=368)	HBsAg^-^ (N=6742)	*P*
**Whole blood counts**			
WBC (10^9^/L)	6.156±2.608	6.564±2.790	**0.001**
Neutrophil (10^9^/L)	3.706±2.285	4.060±2.299	**0.001**
Lymphocyte (10^9^/L)	1.714±0.774	1.756±0.739	0.191
Monocyte (10^9^/L)	0.549±0.357	0.566±0.365	0.505
Eosinophil (10^9^/L)	0.160±0.218	0.165±0.329	**0.025**
Basophil (10^9^/L)	0.027±0.026	0.029±0.338	0.101
RBC (10^12^/L)	4.261±0.585	4.224±0.632	0.388
Platelet (10^9^/L)	217.213±83.052	249.164±87.392	**0.001**
Hb (g/L)	122.185±23.006	120.333±24.010	0.297

WBC: white blood cell; RBC: red blood cell; Hb: hemoglobin.

**Table 3:** Liver function parameters of HBsAg^+^ and HBsAg^-^ cohorts

Factors	HBsAg^+^ (N=368)	HBsAg^-^ (N=6742)	*P*
**Liver function parameters**			
ALT (U/L)	24.782±28.464	17.436±15.525	**0.001**
AST (U/L)	24.221±16.396	19.627±13.550	**0.001**
TBIL (umol/L)	12.349±6.554	11.191±12.145	**0.001**
DBIL (umol/L)	4.227±2.481	4.017±3.103	**0.041**
IBIL (umol/L)	8.187±4.921	7.143±4.449	**0.001**
GGT (U/L)	25.919±35.446	26.153±52.675	0.393
ALP (U/L)	73.682±22.497	75.131±38.434	0.511
ALB (g/L)	38.393±5.160	38.415±5.098	0.964
GLB (g/L)	13.495±13.206	26.440±6.735	**0.001**
Prealbumin (mg/L)	191.311±61.890	210.576±77.299	**0.001**
TG (mmol/L)	1.041±0.477	1.230±0.769	**0.001**
CHOL (mmol/L)	4.461±1.060	4.632±1.123	**0.011**
TBA (umol/L)	6.933±7.871	4.967±5.376	**0.001**
HDL (mmol/L)	1.240±0.356	1.247±1.252	0.69
LDL (mmol/L)	2.647±0.856	2.845±3.775	**0.004**
APRI	0.370±0.635	0.225±0.477	**0.001**

TBIL: Total Bilirubin; DBIL: Direct Bilirubin; IBIL: Indirect Bilirubin; ALT: Alanine Transaminase; AST: Aspartate Transaminase; GGT:Gamma-glutamyltransferase; ALP: Alkaline Phosphatase; ALB: albumin; GLB: globulin; TG: triglyceride; CHOL: cholesterol; TBA: Total Bile Acids; HDL: High Density Lipoprotein; LDL: Low Density Lipoprotein; APRI: Aspartate Aminotransferase-to-platelet Ratio Index.

**Table 4:** Baseline clinicopathological of HBsAg^+^/HBeAg^+^ and HBsAg^+^/HBeAg^-^ cohorts

Factors	HBsAg^+^/HBeAg^+^ (N=69)	HBsAg^+^/HBeAg^-^ (N=296)	*P*
synCRLM (Yes/No)	13/56	30/266	**0.043**
Gender (male/female)	48/21	165/131	**0.036**
Age (years, mean±SD)	54.16±12.71	57.31±11.32	0.066
**Anti-HBV treatment**			
with	2	7	
synCRLM (Yes/No)	0/2	1/6	0.571
W/o	67	289	
synCRLM (Yes/No)	13/54	29/260	0.032
**Primary CRC**			
Tumor size (cm)	4.43±2.27	4.78±1.94	0.100
**Location**			
Colon	27 (39.13%)	99 (33.45%)	
Rectum	20 (28.99%)	108 (36.49%)	0.234
N/A	22 (31.88%)	89 (30.06%)	
**Grade**			
Poorly	11 (15.94%)	48 (16.22%)	
Moderately	50 (72.46%)	204 (68.92%)	
Well	3 (4.35%)	10 (3.38%)	0.935
N/A	5 (7.25%)	34 (11.49%)	
**T stage**			
Tis-T2	10 (14.49%)	44 (14.86%)	
T3- T4	54 (78.26%)	215 (72.64%)	0.069
N/A	5 (7.25%)	37 (12.50%)	
**N stage**			
N0	39 (56.52%)	153 (51.69%)	
N1-N2	24 (34.78%)	102 (34.46%)	0.077
N/A	6 (8.70%)	41 (13.85%)	
CEA	17.47±36.62	31.09±121.36	0.894
CA199	100.22±221.79	45.17±117.24	**0.001**

HBsAg: hepatitis B surface antigen; synCRLM: synchronous colorectal liver metastasis; N/A: not available; Tis: tumor *in situ*; CEA: Carcinoembryonic Antigen; CA199: Carbohydrate Antigen 19-9.

**Table 5:** Complete blood counts of HBsAg^+^/HBeAg^+^ and HBsAg^+^/HBeAg^-^ cohorts

Factors	HBsAg^+^/HBeAg^+^ (N=69)	HBsAg^+^/HBeAg^-^ (N=296)	*P*
**Complete blood counts**			
WBC (10^9^/L)	5.531±1.621	6.290±2.733	0.087
Neutrophil (10^9^/L)	3.097±1.386	3.833±2.404	**0.013**
Lymphocyte (10^9^/L)	1.757±0.653	1.709±0.796	0.375
Monocyte (10^9^/L)	0.494±0.222	0.581±0.385	0.601
Eosinophil (10^9^/L)	0.161±0.121	0.160±0.234	0.071
Basophil (10^9^/L)	0.027±0.019	0.027±0.027	0.349
RBC (10^12^/L)	4.246±0.502	4.265±0.601	0.979
Platelet (10^9^/L)	192.507±96.747	222.932±77.912	**0.002**
Hb (g/L)	126.962±22.316	121.156±23.101	0.071

WBC: white blood cell; RBC: red blood cell; Hb: hemoglobin.

**Table 6:** Liver function parameters of HBsAg^+^/HBeAg^+^ and HBsAg^+^/HBeAg^-^ cohorts

Factors	HBsAg^+^/HBeAg^+^ (N=69)	HBsAg^+^/HBeAg^-^ (N=296)	*P*
**ALT elevation**			
ALT>ULN but <2×ULN	16	30	0.425
ALT≥2×ULN	3	10	
**Liver function**			
ALB (g/L)	37.429±5.566	38.605±5.056	0.095
GLB (g/L)	12.705±13.565	13.540±13.129	0.622
Prealbum (mg/L)	178.213±58.853	193.847±62.448	0.110
TG (mmol/L)	0.937±0.250	1.064±0.511	0.470
CHOL (mmol/L)	4.653±1.262	4.422±1.014	0.206
TBA (umol/L)	8.181±9.225	6.696±7.605	0.279
HDL (mmol/L)	1.203±0.306	1.247±0.366	0.789
LDL (mmol/L)	2.844±1.128	2.608±0.788	0.335
APRI	0.684±1.314	0.294±0.264	**0.001**

TBIL: Total Bilirubin; DBIL: Direct Bilirubin; IBIL: Indirect Bilirubin; ALT: Alanine Transaminase; AST: Aspartate Transaminase; GGT:Gamma-glutamyltransferase; ALP: Alkaline Phosphatase; ALB: albumin; GLB: globulin; TG: triglyceride; CHOL: cholesterol; TBA: Total Bile Acids; HDL: High Density Lipoprotein; LDL: Low Density Lipoprotein; APRI: Aspartate Aminotransferase-to-platelet Ratio Index.

**Table 7:** Univariate logistic regression analysis of the significant predictors for synCRLM

Variable	Coefficient	SE	Wald *x*2	*P*	Odds ratio	95%CI
Gender	-0.223	0.087	6.597	0.010*	0.800	0.675-0.949
Age	-0.132	0.084	2.485	0.115	0.876	0.743-1.033
Glu	-0.074	0.087	0.730	0.393	0.928	0.782-1.101
HBsAg	0.348	0.168	4.296	0.038*	1.417	1.019-1.969
HBeAg	1.072	0.311	11.878	0.001*	2.920	1.588-5.371
WBC	0.461	0.088	27.522	<0.001*	1.585	1.334-1.883
Neutrophil	0.668	0.090	55.371	<0.001*	1.950	1.636-2.325
Lymphocyte	-0.273	0.087	9.788	0.002*	0.761	0.642-0.903
Monocyte	0.519	0.088	34.513	<0.001*	1.680	1.413-1.997
Eosinophils	0.188	0.086	4.753	0.029*	1.207	1.019-1.430
Basophils	-0.186	0.087	4.580	0.032*	0.830	0.700-0.984
RBC	-0.101	0.086	1.378	0.240	0.904	0.763-1.070
Hb	-0.185	0.087	4.527	0.033*	0.831	0.701-0.986
Platelet	0.195	0.086	5.158	0.023*	1.215	1.027-1.437
Neu_pct	0.671	0.090	55.496	<0.001*	1.957	1.640-2.335
Lym_pct	-0.813	0.092	78.365	<0.001*	0.443	0.370-0.531
Mono_pct	0.259	0.087	8.924	0.003*	1.295	1.093-1.535
Eosi_pct	-0.003	0.086	0.001	0.970	0.997	0.842-1.180
Baso_pct	-0.196	0.089	4.838	0.028*	0.822	0.691-0.979
ALT	0.561	0.088	40.644	<0.001*	1.753	1.457-2.083
AST	0.876	0.089	97.180	<0.001*	2.402	2.018-2.859
GGT	1.180	0.097	149.362	<0.001*	3.254	2.693-3.932
ALP	0.789	0.090	76.963	<0.001*	2.201	1.846-2.626
TBIL	0.014	0.086	0.026	0.871	1.014	0.857-1.199
DBIL	-0.039	0.088	0.197	0.657	0.962	0.809-1.143
IBIL	0.019	0.086	0.050	0.823	1.019	0.861-1.206
HDL	-0.200	0.104	3.723	0.054	0.818	0.668-1.003
LDL	0.374	0.105	11.012	0.001*	1.415	1.153-1.736
Prealbumin	-0.472	0.092	26.328	<0.001*	0.624	0.521-0.747
ALB	-0.289	0.087	11.163	0.001*	0.749	0.632-0.887
GLB	0.221	0.086	6.616	0.010*	1.248	1.054-1.477
TG	-0.288	0.104	7.678	0.006*	0.750	0.612-0.919
CHOL	0.230	0.103	4.970	0.026*	1.259	1.028-1.542
TBA	0.105	0.095	1.214	0.271	1.111	0.921-1.339
LDH	1.071	0.128	63.407	<0.001*	2.764	2.152-3.549
NLR	0.709	0.090	61.497	<0.001*	2.032	1.702-2.426
CEA	1.795	0.118	229.703	<0.001*	6.018	4.772-7.590
CA199	1.281	0.104	153.030	<0.001*	3.601	2.939-4.441
Size	0.143	0.112	1.638	0.201	1.154	0.927-1.437
Location	-0.083	0.038	4.679	0.031*	0.921	0.854-0.992
Differentiated	-0.214	0.122	3.073	0.080	0.807	0.636-1.026
T	0.511	0.079	42.130	<0.001*	1.667	1.428-1.945
N	0.417	0.063	43.897	<0.001*	1.518	1.342-1.717
M	2.310	0.091	638.946	<0.001*	10.074	8.422-12.050

GLU: glucose; HBsAg: hepatitis B surface antigen;HBeAg: hepatitis B e antigen; WBC: white blood cell; RBC: red blood cell; Hb: hemoglobin; Neu_pct: Neutrophil percentage; Lym_pct: Lymphocyte percentage; Mono_pct: Monocyte percentage; Eosi_pct: Eosinophils percentge; Baso_pct: Basophils percentage; ALT: Alanine Transaminase; AST: Aspartate Transaminase; GGT: Gamma-glutamyltransferase; ALP: Alkaline Phosphatase; TBIL: Total Bilirubin; DBIL: Direct Bilirubin; IBIL: Indirect Bilirubin; HDL: High Density Lipoprotein; LDL: Low Density Lipoprotein; ALB: albumin; GLB: globulin; TG: triglyceride; CHOL: cholesterol; TBA: Total Bile Acids; LDH: Lactate Dehydrogenase; NLR: Neutrophil to Lymphocyte Ratio; CEA: Carcinoembryonic Antigen; CA199: Carbohydrate Antigen 199.

**Table 8:** Multivariate logistic regression analysis of the predictors for synCRLM with HBeAg

Variable	Coefficient	SE	Wald *x*2	*P*	Odds ratio	95%CI
**HBeAg**	**0.964**	**0.414**	**5.416**	**0.020**	**2.622**	**1.164 to 5.903**
CEA	1.110	0.152	53.258	<0.001	3.034	2.252 to 4.087
ALT	0.242	0.144	2.828	0.093	1.274	0.961 to 1.688
GGT	0.681	0.147	21.455	<0.001	1.975	1.481 to 2.635
Platelet	0.320	0.137	5.488	0.019	1.378	1.054 to 1.801
T	0.221	0.112	3.897	0.048	1.248	1.002 to 1.554
N	0.001	0.091	0.000	0.995	1.001	0.837 to 1.196
M	2.297	0.145	250.733	<0.001	9.941	7.481 to 13.209
Constant	-5.325	0.383	193.781	0.000	0.005	

synCRLM: synchronous colorectal liver metastasis; HBeAg: hepatitis B e antigen; CEA: Carcinoembryonic Antigen; GGT: Gamma-glutamyltransferase; PLT: platelet; ALT: Alanine Transaminase; SE: Standard error; 95%CI: 95% confidence interval.

**Table 9:** Multivariate logistic regression analysis of the predictors for synCRLM with HBsAg

Variable	Coefficient	SE	Wald *x*2	*P*	Odds ratio	95%CI
**HBsAg**	**0.448**	**0.224**	**3.995**	**0.046**	**1.565**	**1.009 to 2.427**
CEA	1.357	0.140	93.580	<0.001	3.885	2.951 to 5.114
ALT	0.281	0.127	4.917	0.027	1.325	1.033 to 1.698
GGT	0.761	0.130	34.463	<0.001	2.140	1.660 to 2.759
Platelet	0.325	0.120	7.293	0.007	1.383	1.093 to 1.751
T	0.274	0.097	7.930	0.005	1.316	1.087 to 1.593
N	0.110	0.077	2.026	0.155	1.116	0.959 to 1.299
M	2.048	0.130	249.717	<0.001	7.753	6.014 to 9.995
Constant	-5.591	0.339	272.254	0.000	0.005	

synCRLM: synchronous colorectal liver metastasis; HBsAg: hepatitis B surface antigen; CEA: Carcinoembryonic Antigen; ALT: Alanine Transaminase; GGT: Gamma-glutamyltransferase; SE: Standard error; 95%CI: 95% confidence interval.

## Abbreviations

CRC: colorectal cancer
CHB: chronic hepatitis B
CRLM: colorectal liver metastasis
HBeAg: hepatitis B surface antigen
HBeAg: hepatitis B e antigen
HBV: hepatitis B virus
synCRLM: synchronous colorectal liver metastasis
AST: aspartate aminotransferase
APRI: aspartate aminotransferase to platelet ratio index
CEA: carcinoembryonic antigen
CA19-9: carbohydrate antigen 19-9
WBC: White blood cell
GLB: globulin
TG: triglyceride
CHOL: cholesterol
LDL: Low Density Lipoprotein
GGT: Gamma-glutamyltransferase
ALP: Alkaline Phosphatase
